# Supplementary material for: Increasing power in the analysis of responder endpoints in rheumatology: a software tutorial
Source: BMC Rheumatol. 2021 Dec 7;5:54. doi: 10.1186/s41927-021-00224-0 (PMC8650391; doi:10.1186/s41927-021-00224-0)
Supplement: Supplementary file 1 — Additional file 1. Further app functionality and computation detail for the AugBin and MultSampSize Shiny applications. [file 41927_2021_224_MOESM1_ESM.docx]

**Increasing power in the analysis of responder endpoints in rheumatology: a software tutorial - *Supplementary Material***

Martina McMenamin, PhD^1,2^, Michael J Grayling, PhD^3^, Anna Berglind, PhD^4^, James MS Wason, PhD^1,3^

1. MRC Biostatistics Unit, University of Cambridge, Cambridge, United Kingdom
2. WHO Collaborating Centre for Infectious Disease Epidemiology and Control, School of Public Health, The University of Hong Kong, Hong Kong Special Administrative Region, China
3. Population Health Sciences Institute, Newcastle University, Newcastle upon Tyne, United Kingdom
4. Late Respiratory & Immunology, Biometrics, BioPharmaceuticals R&D, AstraZeneca, Gothenburg, Sweden
   1. **Additional screenshots of functionality**


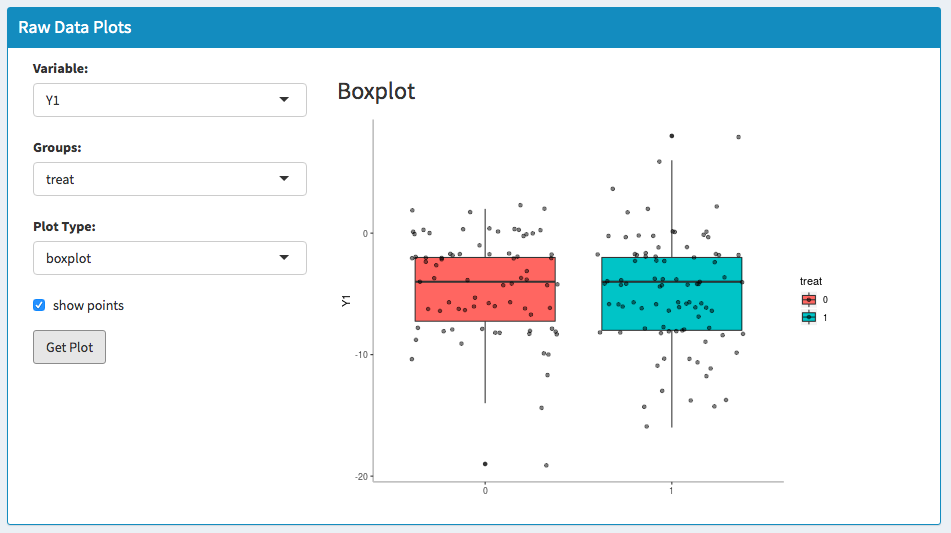

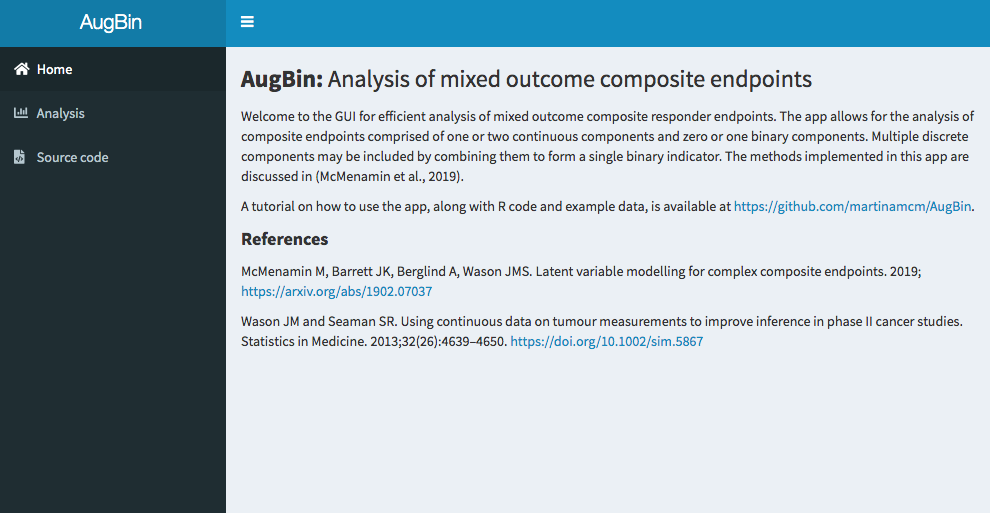


Figure S2 Boxplot for the continuous SLEDAI outcome (Y1) in the MUSE trial, with values shown by the user defined group treatment arm, where 0 refers to the placebo arm and 1 refers to the anifrolumab 300mg arm. ‘Show points’ includes individual data points when checked. The options in the ‘Variable’ and ‘Groups’ dropdown menu will be the column names in the uploaded dataset.

Figure S1 Landing page for the AugBin app which includes the link <https://github.com/martinamcm/AugBin> where users can find a tutorial, R code and example data. Two references are included for users who require more detail on the underlying methods.


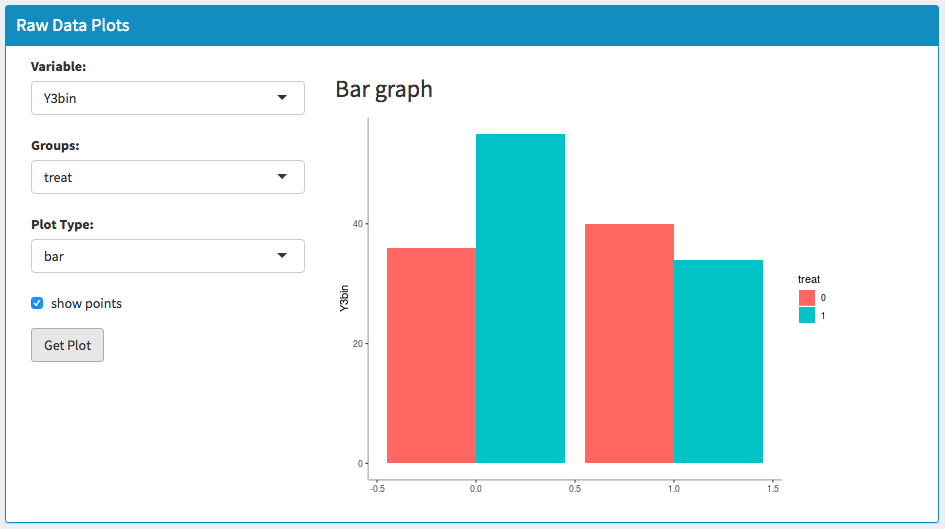

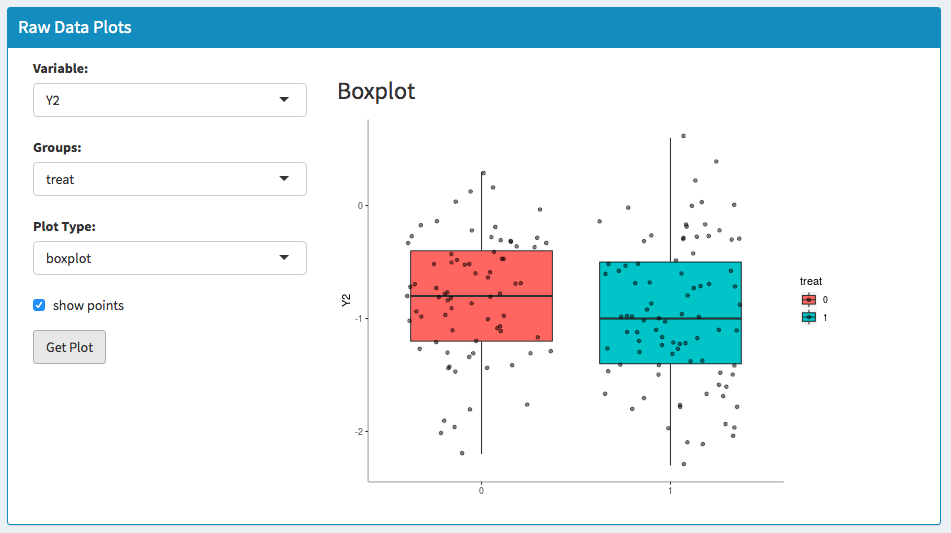


Figure S3 Boxplot for the continuous PGA outcome (Y2) in the MUSE trial, with values shown by the user defined group treatment arm, where 0 refers to the placebo arm and 1 refers to the anifrolumab 300mg arm. ‘Show points’ includes individual data points when checked. The options in the ‘Variable’ and ‘Groups’ dropdown menu will be the column names in the uploaded dataset.

Figure S4 Bar graph of the combined BILAG and taper measures in the MUSE trial (Y3bin) where 0 on the x-axis represents those responding in both the BILAG and taper criteria and 1 indicates those not responding in at least one of the two criteria. The binary response criterion is shown by treatment arm where 0 refers to the placebo arm and 1 refers to the anifrolumab 300mg arm. Options in the ‘Variable’ and ‘Groups’ dropdown menu will be the column names in the uploaded dataset.


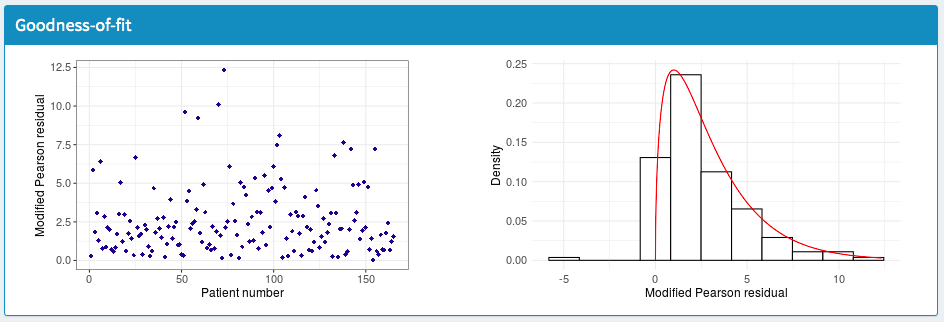

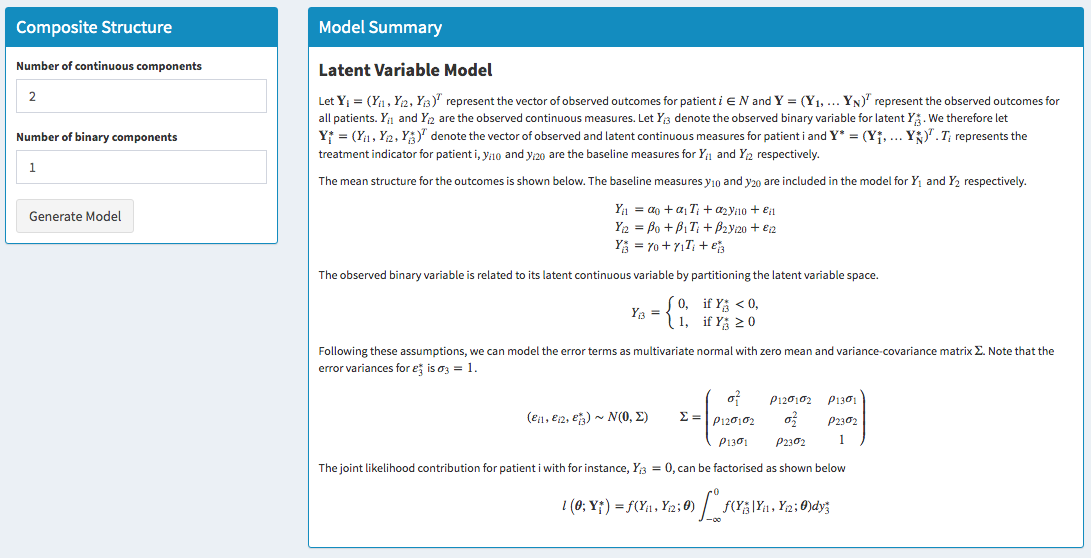


Figure S5 Augmented model for the SRI+OCS endpoint in the MUSE trial. Y_i1_ and Y_i2_ denote the observed values for the continuous SLEDAI and PGA outcomes respectively for patient i, where T_i_ denotes the treatment arm and y_i10_ and y_i20_ are the baseline values. Y_i3_ denotes the indicator for combined BILAG and taper outcomes for patient i, where Y_i3_ equals 0 if the patient has had no Grade A’s and no more than one Grade B in the ordinal BILAG measure **and** sustained reduction of oral corticosteroids (<10mg/day and less than or equal to the dose at week 1 from week 12 through 24); otherwise Y_i3_ equals 1. To fit the model, the measure Y_i3_ is assumed to arise from a latent continuous measure$Y_{i3}^{*}$ where the two quantities are related as shown. The model is fitted by assuming that Y_1_, Y_2_, $Y_{3}^{*}$ follow a multivariate normal distribution with covariance matrix $\Sigma$ as shown, where $\sigma_{1}^{2}$ and $\sigma_{2}^{2}$ denote the variance of Y_1_ and Y_2_ and $\rho_{12},\rho_{13},\rho_{23}$ represents the correlation between the outcomes Y_1,_ Y_2_ $Y_{3}^{*}$. The likelihood is determined as shown from which odds ratios, risk ratios and risk difference effects can be obtained.

Figure S6 Goodness of fit plots for the latent variable method in the MUSE trial dataset. The modified Pearson residuals accounting for correlation between endpoints are shown (left) along with the corresponding density (right) where the model is considered to be a good fit if the residuals follow the chi-squared distribution indicated by the red line.

- 1. **Computational details**

We fit the model in R by coding the likelihood function and probability of response. The bivariate distribution function in the likelihood is estimated using ‘pmvnorm’, applying the method of Genz.^1^ The likelihood maximisation is conducted using a quasi-Newton method based on port routines and can be implemented using the ‘nlminb’ function in the ‘optimx’ package. We use the ‘hessian’ function in the ‘numDeriv’ package to calculate the Hessian matrix using Richardson extrapolation^2^ and obtain the covariance matrix of the model parameters by inverting the Hessian. We ensure finite sample positive definiteness through solving a constrained optimisation problem,^3^ where the nearest correlation matrix projection is used to compute the nearest correlation matrix. We achieve this using the ‘near PD’ function, which implements the algorithm of Higham.^4^ Annotated R code for the analysis is available at the [Github site](https://github.com/martinamcm/AugBin).

**References**

[1] Genz, A. Numerical computation of multivariate normal probabilities. J Comput Graph Stat 1992; 1: 141–150.

[2] Richardson, LF. The approximate arithmetical solution by finite differences of physical problems including differential equations, with an application to the stresses in a masonry dam. Philos Trans R Soc London, Ser A 1911; 210: 307–357.

[3] Fan, J, Liao, Y, Liu, H. An overview of the estimation of large covariance and precision matrices. Econometrics J 2016; 19: C1–C32.

[4] Higham, NJ. Computing the nearest correlation matrix-a problem from finance. IMA J Numer Anal 2002; 22: 329–343.
